# Development of a GFP expression vector for *Cucurbit chlorotic yellows virus*

**DOI:** 10.1186/s12985-018-1004-9

**Published:** 2018-05-24

**Authors:** Ying Wei, Xiaoyu Han, Zhenyue Wang, Qinsheng Gu, Honglian Li, Linlin Chen, Bingjian Sun, Yan Shi

**Affiliations:** 1 College of Plant Protection, Henan Agricultural University, Zhengzhou, 450002 China; 2 Zhengzhou Fruit Research Institute, Chinese Academy of Agriculture Sciences, Zhengzhou, 450009 China

**Keywords:** CCYV, Controller elements, GFP expression

## Abstract

**Background:**

*Cucurbit chlorotic yellows virus* (CCYV), a bipartite crinivirus, causes chlorotic leaf spots and yellowing symptoms on cucurbit leaves. We previously developed an infectious clone of CCYV. Limited work has been conducted on the construction of a crinivirus green fluorescence protein (GFP) expression vector to date.

**Finding:**

We constructed a CCYV GFP expression vector using the “add a gene” strategy based on CCYV RNA2 cDNA constrcut. Three resultant clones, pCCYVGFP_SGC_, pCCYVGFP_CGC_, and pCCYVGFP_CGS,_ were constructed with different promoters used to initiate GFP and CP expression. At 25 dpi GFP fluorescence was detectable not only in leaf veins but also in the surrounding cells. pCCYVGFP_CGC_-infected cucumber leaves exhibited cell spread at 25 dpi, whereas pCCYVGFP_SGC_ and pCCYVGFP_CGS_ were mainly found in single cells. Further observation of pCCYVGFP_CGC_ GFP expression at 30 dpi, 40 dpi, and 50 dpi showed phloem-limited localization in the systemic leaves.

**Conclusions:**

We developed of a CCYV GFP expression vector that will be useful for further study of CCYV movement in cucurbits.

*Cucurbit chlorotic yellows virus* (CCYV), a recently discovered cucurbit-infecting crinivirus in the family *Closteroviridae* [1–7], is among the largest single-strand positive-sense RNA viruses [8]. The bipartite RNA genome comprises a 8607 nucleotide [nt] RNA1 and a 8041-nt RNA2. Recent studies on CCYV [9–12] have been hampered due to lack of reverse genetic tools. The construction of full-length infectious cDNA clones will facilitate the investigation of viral determinants of virus replication and movement, as well as the interactions between viral proteins and host factors. Our previous study developed two sets of full-length CCYV cDNA clones under the control of the T7 RNA polymerase promoter and 35S promoter [13].

Virus-based vectors are useful tools for the study of plant molecular biology. A number of plant virus vectors have been developed to express heterologous genes of interest in plants [14–18]. Only LIYV has been reported to construct a green fluorescence protein (GFP) vector for crinivirus using the ORF fusion strategy. Part of LIYV RNA1 encoded P34 protein was replaced by a GFP gene and further observation of *Nicotiana benthamiana* (Nb) leaves agro-infiltrated by the resultant clone displayed occasional single cells green fluorescence. Limited GFP expression has been observed in *Nicotiana benthamiana* plants, but in no other hosts. Cucumber is an economically important cultivated plant, and the available GFP expression vector of cucurbit viruses is limited [19]. In this study, we constructed a CCYV GFP expression vector using the “add a gene” strategy with an extra subgenomic RNA and a foreign GFP protein, according to a previous study of the closterovirus *Citrus tristeza virus* [20], which suggested that this strategy would be more appropriate than ORF substitution or ORF fusion. We examined the systemic leaves of cucumber plants regarding the resulting GFP fluorescence.

We chose to insert gfp ORF before the CP gene, because the CP subgenomic controller elements (CEs) are more suitable than other CEs [20–23]. The full-length CCYV RNA2 clone was modified to accommodate the *gfp* gene immediately upstream of the CP open reading frame via overlapping polymerase chain reaction (PCR). *Sweet potato chlorotic stunt virus* (SPCSV) CP CEs were used to direct the expression of the CCYV CP gene or GFP. The strategy used to construct the full-length cDNA clones of RNA1 and RNA2 is outlined in Fig. 1. The resulting clones were named pCCYVGFP_SGC_, pCCYVGFP_CGC_, and pCCYVGFP_CGS_. To construct the clone pCCYVGFP_CGS,_ four overlapping fragmens were amplified using the primer pairs showed in Table 1 and the recombinant fragment were obtained using overlap PCR. Restriction enzymes *Mlu*I and *Nru*I were used to insert the fragment into the pCBCCYVRNA2 to acquire the GFP-tagged vector pCCYVGFP_CGS._ Based on pCCYVGFP_CGS,_ a fragment from 4811 to 5193 nt covering CCYVCP CEs and partial CP sequence was amplified using primer pair CCYVCcgccF2F/CCYVCcgscF2R. pCCYVGFP_CGS_ and the fragment were digested by PacI and NruI to acquire the recombinant clone pCCYVGFP_CGC._ pCCYVGFP_SGC_ vector was cloned based on pCCYVGFP_CGC._ Three overlapping fragments were amplified using primer pairs CCYV_cgsc_F1F/CCYVsgccF1R, CCYVsgccSPCSVCPpF/CCYVsgccSPCSVCPpR, and CCYVsgccGFPF/CCYVcgscF2R, and overlap PCR was used to ligate the fragments and inserted into pCBCCYVRNA2 vector using restriction enzymes *Mlu*I and *Nru*I. The resultant clones were transformed into *Agrobacterium tumefaciens *strain GV3101 for agroinfiltration. At 25 days after agroinfiltration of pCCYVGFP with pCBCCYVRNA1 and pCBP1/HC-Pro in *C. sativus*, the adaxial side of leaf was used to observe the GFP fluorescence using epifluorescence microscope (Nikon ECLIPSE Ti-S) with the excitation of 460-480 nm and emission wavelength of 500-540 nm, respectively (Fig. 2a). GFP fluorescence was detectable not only in leaf veins, but also in the surrounding cells. This result is consistent with that of a previous study of CCYV localization in leaf laminae using immunoblots [9]. We observed that pCCYVGFP_CGC_-inoculated cucumber leaves exhibited cell spread at 25 dpi, whereas the other two constructs were mainly found in single cells (Fig. 2b). The number of pCCYVGFP_CGC_ fluorescent cells was significantly higher than that of pCCYVGFP_CGS_ fluorescent cells. Further observation of pCCYVGFP_CGC_ at 30 dpi showed that GFP was mainly vein-limited in cucumber systemic leaves (Fig. 3). Time course observation of pCCYVGFP_CGC_ GFP fluorescence showed that at 40 dpi the GFP fluorescence was mainly focused in the second systemic leaf and at 50 dpi the fluorescence was mainly focused in the main vein of systemic leafs showing the most fluorescence in the third systemic leaf (Fig. 3).

**Fig. 1:**
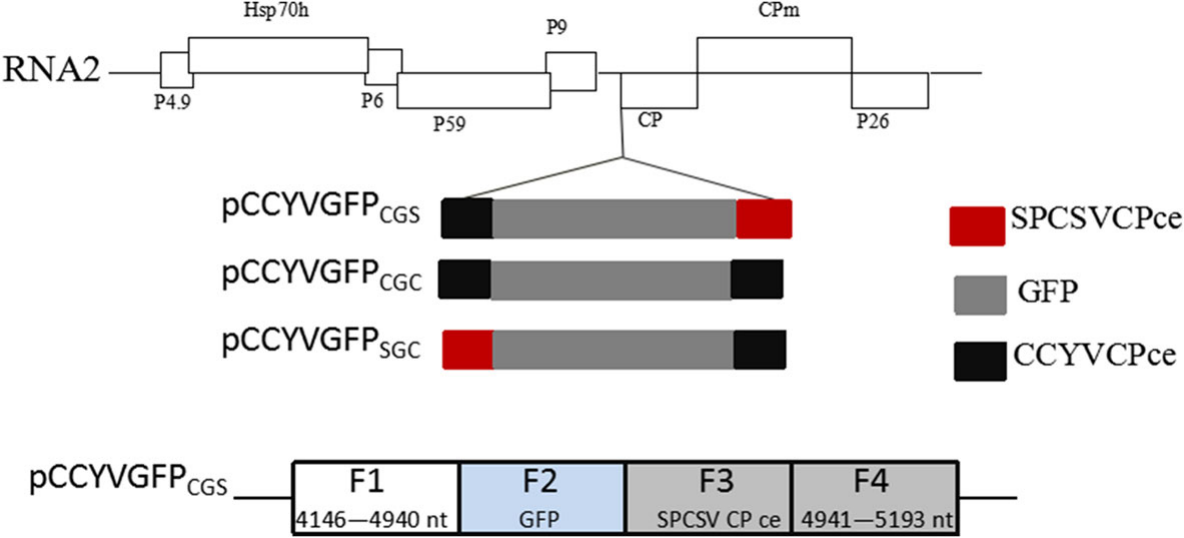
Schematic representation of the construction of a green fluorescent protein (GFP) expression vector of *Cucurbit chlorotic yellows virus* (CCYV). GFP was inserted immediately upstream of the CP gene. Three different combinations of CCYV CP controller elements (CEs) and *Sweet potato chlorotic stunt virus* (SPCSV) CP CEs are shown. Red square indicates CEs of SPCSVCP; black square indicates CEs of CCYVCP. Grey square indicates the *gfp* gene

**Table 1:** Primer sets used in the paper

Primer name	Sequence (5′-3′)
CCYVcgscF1F	CTACTATTGGACGCGTTATTG
CCYVcgscF1GFPR	CTCGCCCTTGCTCACCATATTTAATGTAGATCGAGT
CCYVcgscGFPF	ACTCGATCTACATTAAATATGGTGAGCAAGGGCGAG
CCYVcgscGFPR	ATACCAAGTTTTAATTAATCAAAGATCTACCATGTACAGC
CCYVcgscSPCSVCPpF	AGATCTTTGATTAATTAAAACTTGGTATCGCGGTTG
CCYVcgscSPCSVpCPR	ATTGTCAGTCTTCTCCATACTCGTCTCACTGCTTAG
CCYVcgscF2F	CTAAGCAGTGAGACGAGTATGGAGAAGACTGACAAT
CCYVcgscF2R	CGAAATCCCTCATACACTGTTC
CCYVCcgccF2F	CGCTTAATTAATCAGTGATTATCTTCAAATTC
CCYVsgccF1R	ACCAACCGCGATACCAAGTTTCAGTTAAAAAATTTTGGTA
CCYVsgccSPCSVCPpF	TACCAAAATTTTTTAACTGAAACTTGGTATCGCGGTTGGT
CCYVsgccSPCSVCPpR	CCTCGCCCTTGCTCACCATACTCGTCTCACTGCTTAGTT
CCYVsgccGFPF	AACTAAGCAGTGAGACGAGTATGGTGAGCAAGGGCGAGG

**Fig. 2:**
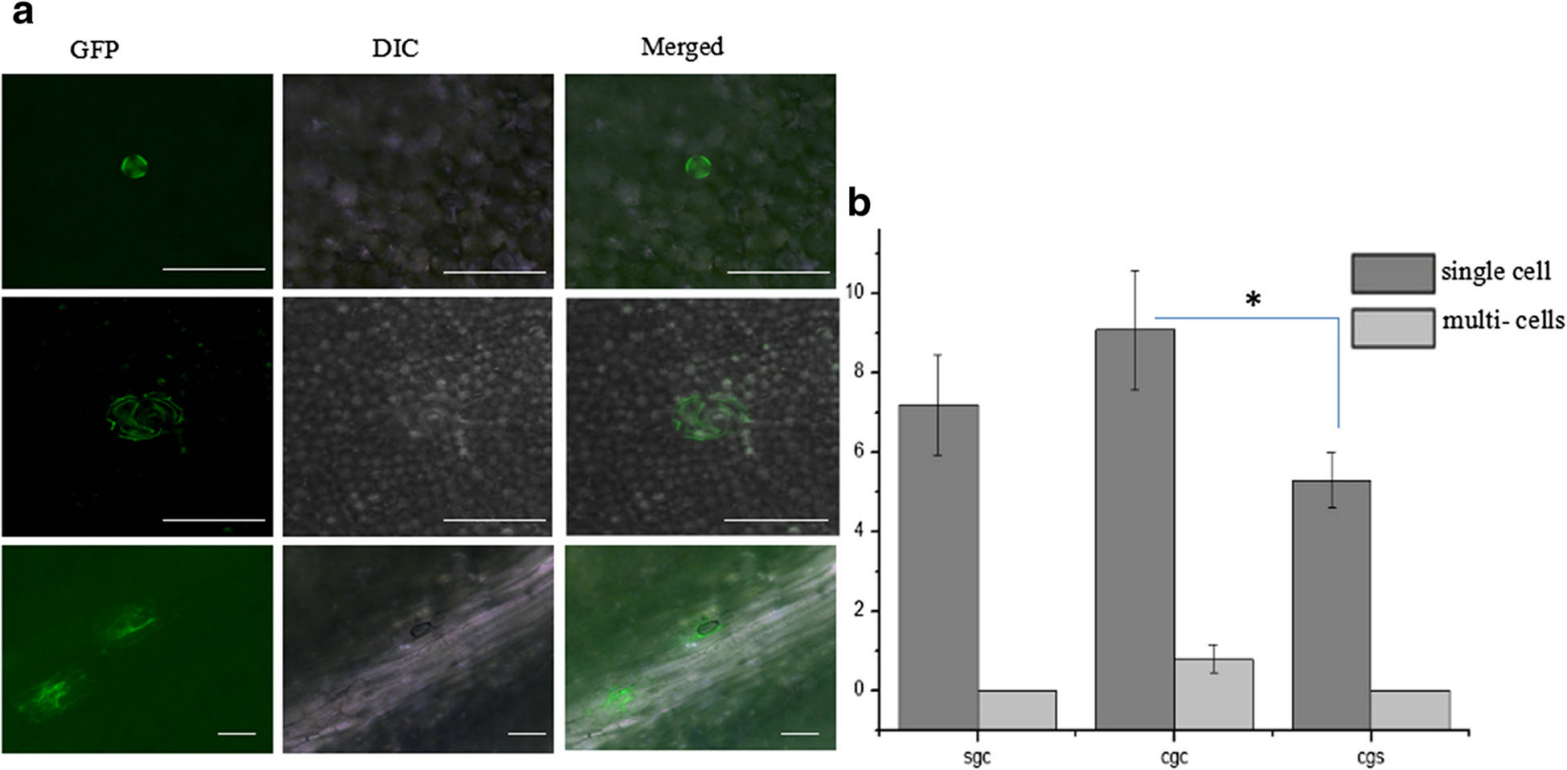
CCYV GFP expression in the systemic leaves of cucumber at 25 dpi. **a** From left to right: GFP expression in the leaf vein, GFP expression in a single cell beside the leaf vein, and GFP expression in the surrounding cells. Bars represent 200 μm. **b** Counting expression spots of the different constructs on systemic leaves. We selected 10 different visual fields for counting. Dark grey bars indicates the single fluorescent cells and light grey bars indicates the multiple cells showing fluorescence. Sgc, cgc, cgs represents the three different strategies used for GFP expression. * indicates significant difference with *P* value<0.05

**Fig. 3:**
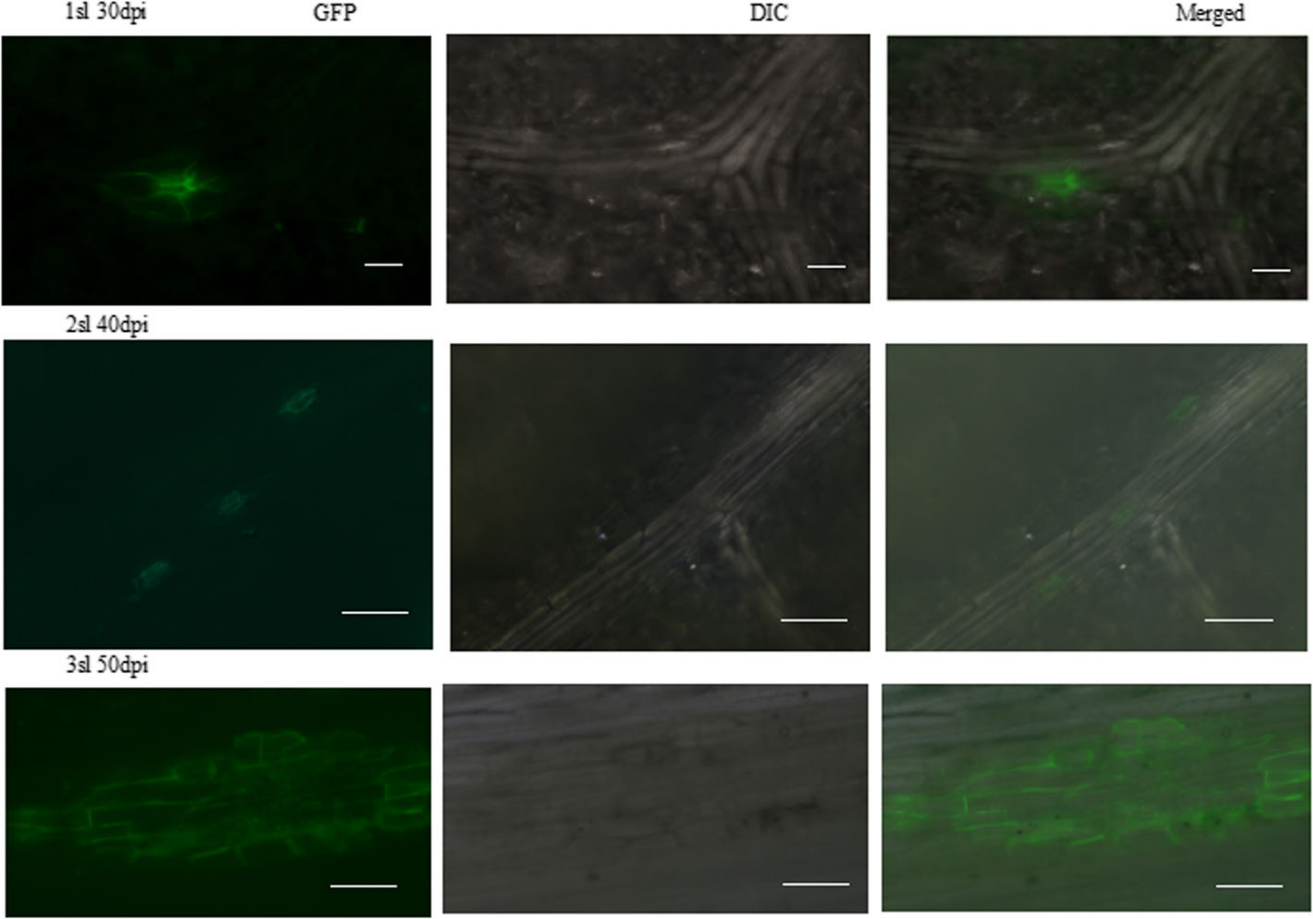
Time course observation of pCCYVGFP_CGC_ GFP expression at 30dpi, 40dpi and 50dpi. Photos were taken under epifluorescence. Bars represent 200 μm. A differential interference contrast (DIC) image was shown in each image.

As previously reported for the closterovirus CTV, a heterologous BYV sgRNA CE to control GFP expression was stable and worked best of the stratefies examined [20]. The closterovirus beet yellow stunt virus (BYSV) 248 bp upstream of the CP coding region was chosen to direct the expression of BYV CP, and the original CP promoter was used to direct GFP expression [24]. We chose a 201-bp CE sequence upstream of the SPCSV CP coding region to test the heterologous effect, and a 131-bp CE of CCYV CP to test the homologous effect. According to our results, the homologous sequence repeats in the vector promoted the GFP expression of CCYV, whereas when we used the CE of SPCSV CP to direct expression of CCYV CP or GFP, GFP expression was delayed. Hence, pCCYVGFP_CGC_ can be used as a candidate for further study of GFP expression. Here we tested the insertion of GFP before the CP coding region. Other positions could be tested further to see if the other position would work better.

## Abbreviations

CCYV: *Cucurbit chlorotic yellows virus*
CEs: controller elements
*GFP*: green fluorescent protein
SPCSV: *Sweet potato chlorotic stunt virus*
